# Supplementary material for: Comparison of national and international sedentary behaviour and physical activity guidelines for older adults: A systematic review and quality appraisal with AGREE II
Source: PLoS One. 2023 Nov 27;18(11):e0294784. doi: 10.1371/journal.pone.0294784 (PMC10681178; doi:10.1371/journal.pone.0294784)
Supplement: S1 Table — (PDF) [file pone.0294784.s002.pdf]

S1 Table. Search Strategy.

**Database: OVID Medline Epub Ahead of Print, In-Process & Other Non-Indexed Citations, Ovid MEDLINE(R) Daily and Ovid MEDLINE(R) 1946 to Present**	
**1**	Sedentary Behavior/ (13463)
**2**	(sedentary lifestyle* or sedentary life style* or sedentary time* or sedentary behavio?r* or physical inactivit*).ti,ab,kf. (26976)
**3**	exp Overweight/ (269121)
**4**	(obes* or overweight*).ti,ab,kf. (400758)
**5**	exp Exercise/ (244224)
**6**	(exercis* or physical activit*).ti,ab,kf. (471646)
**7**	or/1-6 (958069)
**8**	exp guideline/ (37617)
**9**	guideline*.pt. (16562)
**10**	practice guideline*.pt. (30368)
**11**	exp Consensus Development Conference/ (12653)
**12**	exp Consensus Development Conference/ (12653)
**13**	or/8-12 (47511)
**14**	7 and 13 (2030)
**15**	exp Aged/ (3445994)
**16**	(older adult* or senior* or elder*).ti,ab,kf. (450501)
**17**	15 or 16 (3590530)
**18**	14 and 17 (232)
**Database: Embase <1974 to 2023 May 01>**	
**1**	exp sedentary lifestyle/ (20096)
**2**	(sedentary behavio?r* or sedentary lifestyle* or sedentary life style* or physical inactivit*).ti,ab,kw. (33701)
**3**	exp obesity/ (650887)
**4**	(obes* or overweight*).ti,ab,kw. (601263)
**5**	exp exercise/ (425711)
**6**	exp physical activity/ (527311)
**7**	(exercis* or physical activit*).ti,ab,kw. (636384)
**8**	or/1-7 (1737944)
**9**	exp aged/ (3578983)
**10**	(older adult* or senior* or elder*).ti,ab,kw. (629916)
**11**	exp practice guideline/ (707155)
**12**	exp consensus development/ (27341)
**13**	consensus development.pt. (0)
**14**	(consensus development or practice guideline*).ti,ab. (47586)
**15**	9 or 10 (3765054)
**16**	11 or 12 or 13 or 14 (720227)
**17**	8 and 15 and 16 (6996)
**18**	(review or preprint).pt. (3152027)
**19**	17 not 18 (6189)
**Database: Global Health <1910 to 2023 Week 17>**	
**1**	exp obesity/ (151511)

**2**	exp exercise/ (38706)
**3**	exp physical activity/ (84286)
**4**	sedentary behaviour/ (990)
**5**	(sedentary behavio?r* or sedentary lifestyle* or sedentary life style* or physical inactivit*).mp. (11479)
**6**	(obes* or overweight* or exercis* or physical activit*).mp. (271477)
**7**	or/1-6 (273430)
**8**	elderly/ (72373)
**9**	(older adult* or senior* or elder*).mp. (102000)
**10**	8 or 9 (102000)
**11**	7 and 10 (16198)
**12**	guidelines/ (67560)
**13**	(guideline* or consensus development).mp. (119621)
**14**	12 or 13 (119621)
**15**	11 and 14 (1010)
**Database: Web of Science**	
**1**	TS=((sedentary lifestyle* or sedentary life style* or sedentary time* or sedentary behavio?r* or physical inactivit* or obes* or overweight* or exercise* or physical activit* or physical inactivit*))
**2**	TS=((guideline* or consensus development conference*))
**3**	#2 AND #1
**4**	TS=((older adult* or senior*or elder*))
**5**	#4 AND #3
**Database: CINHAL**	
**S1**	(MH "Life Style, Sedentary+")
**S2**	MH "Automobile Driving" OR MH "Television" OR "video gaming" OR "video game" OR "computer gaming" OR "video game time" OR "computer game" OR MH "Computers and Computerization Utilization" OR MH "Video Games" OR "screen time" OR "screen entertainment" OR "screen behavior" OR "screen behavior" OR "screen-based entertainment" OR "television viewing" OR "television time" OR "TV viewing" OR "TV watching" OR "TV time" OR "watch television" OR "view television" OR "watchTV" OR "screen watching" OR "screen time" OR "screen entertainment" OR "screen behavior" OR "screen behavior" OR "screen-based entertainment" OR "sedentary behavior" OR "sedentary behavior" OR "sedentary lifestyle" OR "prolonged sitting" OR "sitting time" OR "lying time" OR MH "Life Style, Sedentary+"
**S3**	(MH "Obesity+")
**S4**	(obes* or overweight)
**S5**	(MH "Exercise+")
**S6**	S1 OR S2 OR S3 OR S4 OR S5 OR S6
**S7**	(MH "Practice Guidelines")
**S9**	(MH "Consensus")
**S10**	PT practice guidelines
**S11**	S8 OR S9 OR S10
**S12**	S7 AND S11

**S13**	(MH “Aged+”)
**S14**	(older adult* or senior* or elder*)
**S15**	S13 OR S14
**S16**	S12 AND S15
