# Supplementary material for: Comparison of national and international sedentary behaviour and physical activity guidelines for older adults: A systematic review and quality appraisal with AGREE II
Source: PLoS One. 2023 Nov 27;18(11):e0294784. doi: 10.1371/journal.pone.0294784 (PMC10681178; doi:10.1371/journal.pone.0294784)
Supplement: S2 Table — (DOCX) [file pone.0294784.s003.docx]

**S2 Table: Summary of the sedentary behaviour guidelines**

| **Sedentary Behaviour guideline [country, year]** | **Stakeholders** | **Conflict of interest** | **Type of study** | **Definition** | **Recommendation [certainty of evidence]** | **Funding** |
| --- | --- | --- | --- | --- | --- | --- |
| Physical Activity Guidelines for the Brazilian Population  [Brazil, 2022] [10] | - Researchers - Patient partners | N/A | - Opinions of stakeholders - Other guidelines (Argentina, Uruguay, Latin America, WHO) | Awake, sitting, reclining, or lying down and spending little amounts of energy | Every hour of sitting, stand up and stretch for 5 minutes  [N/A] | Ministry of Health of Brazil |
| Canadian 24-Hour Movement Guidelines for Adults aged 18–64 years and Adults aged 65 years or older  [Canada, 2020] [12] | - Researchers - Clinicians - Patient partners - Organizations - Knowledge managers - Government stakeholders - Reference librarian | Yes | - Overview of reviews - Opinions of stakeholders - Other guidelines (USA) | Any awake behavior characterized by an energy expenditure ≤1.5 METs while sitting, reclining, or lying posture | Limiting sedentary time to < 8 hours/day and ≤3 hours/day of recreational screen time [Low, GRADE]  Breaking up long periods of sitting as often as possible [Very Low, GRADE] | Public Health Agency of Canada, the Canadian Society for Exercise Physiology, Queen’s University, and ParticipACTION |
| National Physical Activity Guidelines for Japan  [Japan, 2013], [21] | - Researchers | N/A | N/A | <5,000 steps/day (‘sedentary’) | Reduce sitting time. [N/A] | Ministry of Health, Labour, Welfare |
| The 2017 Dutch Physical Activity Guidelines [Netherlands, 2017], [22] | - Researchers - Clinicians - Government stakeholders | Yes | N/A | Any awake behaviour with low energy expenditure ≤1.5 METs while sitting, reclining, or lying | Avoid long periods sitting down [N/A] | N/A |
| Guidelines on Physical Activity for Older People (aged 65 years and over) [New Zealand, 2013], [18] | - Researchers - Clinicians - Patient partners - Organizations - Government stakeholders | N/A | - Narrative reviews - Two governmental reports from USA | Activities that require little or no energy expenditure (≤1.5 MET) | Limit sedentary behaviour and be as physically active as possible [Level III-2, NHMRC] | Ministry of Health |
| Qatar National Physical Activity Guidelines 2^nd^ Edition [Qatar, 2021], [23] | - Researchers - Clinicians - Government stakeholders | N/A | N/A | Any awake behaviour with low energy expenditure ≤1.5 METs while sitting, reclining, or lying | Reduce total time spent sitting during waking hours and take regular breaks every 20-30 minutes, especially with screen-based activities. Make physical activity a daily habit to reduce sitting time (e.g., using stairs, active commuting, standing, walking, bodyweight resistance exercises) [N/A] | N/A |
| 24-hr Movement Practice Guidelines for Saudi Arabia [ Saudi Arabia, 2021], [19] | - Researchers - Clinicians | N/A | - Other guidelines in individuals <65 years (WHO [2020], UK [2019], Canada [2017], Australian [2012]) | Any awake behavior characterized by an energy expenditure ≤1.5 METs while sitting, reclining, or lying posture | Reduce amount of time in prolonged sitting throughout the day. Break up long periods of sitting as often as possible with at least light physical activity, when physically possible, or standing [N/A] | N/A |
| Physical Activity Guidelines for Americans 2nd edition  [USA, 2018], [11] | - Researchers - Clinicians - Government stakeholders | Yes | - Overview of reviews - Opinions of stakeholders - Other guidelines (USA [2008, 2014], Canada) | Any awake behavior characterized by an energy expenditure ≤1.5 METs while sitting, reclining, or lying posture | Adults should move more and sit less throughout the day. Some physical activity is better than none [Low, GRADE] | U.S. Department of Health and Human Services |
| Physical activity guidelines for older adults  [UK, 2022], [20] | - Researchers - Clinicians - Patient partners - Organizations - Knowledge managers - Government stakeholders - Reference Librarian | N/A | - Critical review - Cohort studies | Sitting, reclining, or lying posture during awaking hours, undertaking little movement or activity and using little energy above what is used at rest. | Break up prolonged sedentary time with light activity when physically possible, or at least with standing. Avoid long periods of screen time, stand up and move during TV advertisement breaks, stand, or walk while on the phone, use the stairs, when possible, take up active hobbies [N/A] | Centre for Exercise, Nutrition and Health Sciences, School for Policy Studies at the University of Bristol |
| World Health Organization 2020 guidelines on physical activity and sedentary behaviour  [WHO, 2020], [9] | - Researchers - Clinicians - Patient partners - Organizations - Government stakeholders | Yes | - Systematic review - Other guidelines (Australian, Canada, USA) | Any awake behavior characterized by an energy expenditure ≤1.5 METs while sitting, reclining, or lying posture | Adults should limit the amount of time being sedentary. Replacing sedentary time with physical activity of any intensity (including light intensity) [Low, GRADE] | The Public Health Agency of Canada and the Government of Norway |

N/A = Not Available, METs = metabolic equivalents, GRADE = Grading of Recommendations, Assessment, Development, and Evaluations
