# Supplementary material for: Comparison of national and international sedentary behaviour and physical activity guidelines for older adults: A systematic review and quality appraisal with AGREE II
Source: PLoS One. 2023 Nov 27;18(11):e0294784. doi: 10.1371/journal.pone.0294784 (PMC10681178; doi:10.1371/journal.pone.0294784)
Supplement: S3 Table — (DOCX) [file pone.0294784.s004.docx]

**S3 Table: Summary of the physical activity guidelines**

| **Physical Activity Guideline [Country, Year]** | **Stakeholders** | **Conflict of interest** | **Type of evidence** | **Aerobic training [certainty of evidence]** | **Strength training [certainty of evidence]** | **Balance training [certainty of evidence]** | **Flexibility training [certainty of evidence]** | **Funding source** |
| --- | --- | --- | --- | --- | --- | --- | --- | --- |
| National Physical Activity Recommendations for Older Australians: Discussion Document [Australia, 2009],[27] | - Researchers - Clinicians - Patient partners - Organizations - Knowledge managers - Government stakeholders | N/A | - Systematic reviews - RCTs | 5 -7 days/week ≥30 mins.  12 – 14 RPE; 40 – 60% HR max [moderate]  Increase duration (≥ 30 min) before increasing intensity up to moderate. | 2 – 3 days/week at progressive overload, 2-3 sets, 10-12 repetitions  4 upper (biceps, shoulder flexion, chest press, back row) and 4 lower body (hamstrings, quadriceps, leg press, calves) [moderate, NHMRC] | 1 -7 days/week at progressive overload [moderate]  Dynamic = focus on mobility; static = focus on 1-leg stance | 2-3 days/week, 10-30 seconds,  3-4 times for each stretch. [low]  Chest, neck, hands, triceps, hamstrings, quadriceps, hip flexors, calf soleus, gastrocnemius. | National Ageing Research Institute |
| Austrian Physical Activity Recommendations – Key Messages [Austria, 2020] ,[28] | - Researchers | N/A | - N/A | Moderate or vigorous intensity for 150-300 minutes of moderate, or 75-150 of vigorous [moderate] | ≥2 days/week  (Intensity and volume N/A) [N/A] | N/A | N/A | Austrian National Public Health Institute |
| Physical Activity Guidelines for the Brazilian Population: Recommendations Report [Brazil, 2022] ,[10] | - Researchers - Patient partners | N/A | - Systematic review - Opinions of stakeholders - Focus groups | Moderate to vigorous,150 minutes of moderate intensity or 75 minutes of vigorous intensity each week [N/A] | ≥2 days/week  moderate to vigorous intensity  (Volume N/A) [N/A] | N/A | N/A | Ministry of Health of Brazil |
| Canadian 24-Hour Movement Guidelines for Adults aged18–64 years and Adults aged 65 years or older: [Canada, 2020] ,[12] | - Researchers - Clinicians - Patient partners - Organizations - Knowledge managers - Government stakeholders - Reference librarian | Yes | - Overview of reviews - Systematic review - RCTs | Moderate to vigorous intensity for at least 150 minutes/week [moderate to high, GRADE] | 2 days/week of major muscle groups (Intensity and volume N/A) [moderate to high, GRADE] | Physical activities that challenge balance. (Frequency, intensity and volume N/A) [moderate to high, GRADE] | N/A | Public Health Agency of Canada, Canadian Society for Exercise Physiology, Queen’s University, and ParticipACTION |
| Physical Activity Guidelines for Chinese [China, 2021] ,[31] | - Researchers - Organizations - Government stakeholders | Yes | - Other countries guidelines | 150-300 minutes of moderate intensity, 75-100 minutes of vigorous intensity per week [N/A] | 2 days/week  (Intensity and volume N/A) [N/A] | Mentioned but not in detail. [N/A] | Mentioned but not in detail. [N/A] | Key Program of Social Sciences of Jiangsu Province |
| Copenhagen Consensus statement 2019: physical activity and ageing [Denmark, 2019] ,[32] | - Researchers | N/A | - Opinions of stakeholders - Cohort - RCTs | Moderate to vigorous intensity, ≥30 minutes/day [N/A] | N/A | N/A | N/A | N/A |
| The German recommendations for physical activity promotion [German, 2019] ,[25] | - Researchers - Government stakeholders - Reference librarian | N/A | - Systematic review - Opinions of stakeholders | 5 days/week, moderate intensity ≥  10 minutes distributed over each day, with weekly total 150 minutes. [N/A] | 2 days/week (Intensity and volume N/A) [N/A] | N/A | N/A | German Federal Ministry of Health |
| Consensus Physical Activity Guidelines for Asian Indians [India, 2012] ,[24] | - Researchers - Clinicians | Yes | - Systematic review - Cohort - RCT | Moderate intensity ≥30 minutes/day, bouts of at least 10 min in duration. [N/A] | 2 days/week  (Intensity and volume N/A) [N/A] | N/A | N/A | N/A |
| National Physical Activity Guidelines for Japan [Japan, 2013] ,[21] | - Researchers | N/A | N/A | Low to moderate intensity, 40 minutes/day or 8,000–10,000 steps/day [N/A] | N/A | N/A | N/A | Ministry of Health, Labour, Welfare |
| The 2017 Dutch Physical Activity Guidelines [Netherlands, 2017] ,[22] | - Researchers - Clinicians - Government stakeholders | Yes | - Systematic review - Cohort - RCTs | Few days/week at moderate intensity for  150 minutes/week [N/A] | 2 days/week  (Intensity and volume N/A) [N/A] | ≥2 days/week  (Intensity and volume N/A) [N/A] | N/A | N/A |
| Guidelines on Physical Activity for Older People (aged 65 years and over) [New Zealand, 2013] ,[18] | - Researchers - Clinicians - Patient partners - Organizations - Government stakeholders | N/A | - Systematic reviews - Literature reviews - Cohort - RCTs | 30 minutes/day, 5 days/week. Vigorous intensity 15 minutes/day, 5 days/week. Moderate-to-vigorous 10 minutes/day, 5 days/week.  150 minutes of moderate activity, or 75 minutes of vigorous activity [Level I and II, NHMRC] | ≥2 days/week (Intensity and volume N/A) [Level I and II, NHMRC] | 3 sessions/ week (Intensity and volume N/A) [Level I and II, NHMRC] | 3 sessions/ week (Intensity and volume N/A) [Level I and II, NHMRC ] | Ministry of Health |
| The action plan on physical activity: Working together for physical activity [Norway, 2001] ,[29] | - Researchers - Government stakeholders | N/A | N/A | moderate to high intensity ≥30 minutes/day  Activities may be split up in shorter periods (10 minutes over the course of the day) [N/A] | N/A | N/A | N/A | N/A |
| Polish Forum for Prevention Guidelines on physical activity [Poland, 2009] ,[30] | - Researchers - Clinicians | N/A | N/A | ≥3 days/week, moderate intensity (60-75% of max HR) for 20 – 60 minutes. [N/A] | ≥10-15% 1RM intensity (Frequency and volume N/A) [N/A] | N/A | N/A | N/A |
| Qatar National Physical Activity Guidelines 2^nd^ Edition [Qatar, 2021] ,[23] | - Researchers - Clinicians - Government stakeholders | N/A | N/A | Cardiovascular endurance activities involve large muscle groups ≥ 5 days per week (moderate) or ≥ 3 days per week (vigorous) or 3-5 days per week (combination of moderate and vigorous) [N/A] | 8-10 compound exercises target major muscle groups. ≥ 2 days/week with 48 hours rest for same muscle groups at light (40-50% 1 RM) or moderate (60-70% 1 RM) intensity. 8-12 reps/set of exercise, 1-2 sets of each exercise. Moderate speed movements (6 sec/rep), 2-3 min rest between sets [N/A] | Individuals at risk of falls should incorporate balance exercises ≥ 2 days/week [N/A] | Stretch major muscle groups 2-3 days/week, each stretch should be held for 10-30 sec to the point of tightness or slight discomfort. Repeat each stretch 2-4 times (accumulating 60 sec/ flexibility exercise) [N/A] | N/A |
| 24-hr Movement Practice Guidelines for Saudi Arabia [ Saudi Arabia, 2021] ,[19] | - Researchers - Clinicians | N/A | - Other guidelines in individuals (WHO [2020], UK [2019], Canada [2017], Australian [2012]) | 150 mins of moderate—intensity/week or 75 mins of vigorous-intensity/week, or an equivalence combination of moderate-to-vigorous aerobic physical activity spread throughout the week [Strong, based on expert opinion] | Muscle strength minimum of 2 days/week [Strong, based on expert opinion] | Balance training of 2 days/week [Strong, based on expert opinion] | Flexibility training of 2 days/week [Strong, based on expert opinion] | N/A |
| Physical activity guidelines for older adults [UK, 2022] ,[20] | - Researchers - Clinicians - Patient partners - Organizations - Knowledge managers - Government stakeholders - Reference Librarian | N/A | - Systematic reviews - Cohort - RCTs - Other guidelines | 150 minutes of moderate or 75 minutes of vigorous activity each week [N/A] | ≥2 days/week (Intensity and volume N/A) [N/A] | ≥2 days/week  (Intensity and volume N/A) [N/A] | ≥2 days/week  (Intensity and volume N/A) [N/A] | Centre for Exercise, Nutrition and Health Sciences, School for Policy Studies at the University of Bristol |
| Physical Activity Guidelines for Americans 2nd edition [USA, 2018] ,[11] | - Researchers - Clinicians - Government stakeholders | Yes | - Systematic review - Cohort - RCT | 150 to 300 minutes of moderate-intensity physical activity a week, or an equivalent amount (75 to 150 minutes) of vigorous-intensity activity per week [moderate to high, GRADE] | 2 days/week, moderate or high intensity (Volume N/A) [moderate to high, GRADE] | Mentioned but not in detail. [N/A] | N/A | U.S. Department of Health and Human Services |
| World Health Organization 2020 guidelines on physical activity and sedentary behaviour [WHO, 2020] ,[9] | - Researchers - Clinicians - Patient partners - Organizations - Government stakeholders | Yes | - Systematic review | Moderate to vigorous,150–300 minutes of moderate intensity or 75–150 minutes of vigorous intensity each week [moderate, GRADE] | 3 days/week, moderate or high intensity  (Volume N/A) [moderate, GRADE] | 3 days/week, moderate or high intensity (Volume N/A) [moderate, GRADE] | 3 days/week, moderate or high intensity (Volume N/A) [N/A] | The Public Health Agency of Canada and the Government of Norway |

N/A = Not Available, RCT = Randomized Controlled Trial, RPE = Borg Rating of Perceived Exertion, HR = Heart Rate, GRADE = Grading of Recommendations, Assessment, Development, and Evaluations, NHMRC = National Health and Medical Research Council
