# Supplementary material for: Comparison of national and international sedentary behaviour and physical activity guidelines for older adults: A systematic review and quality appraisal with AGREE II
Source: PLoS One. 2023 Nov 27;18(11):e0294784. doi: 10.1371/journal.pone.0294784 (PMC10681178; doi:10.1371/journal.pone.0294784)
Supplement: Attachment — Submitted filename: Response to Reviewers and Editor.pdf [file pone.0294784.s006.pdf]

Thank you for reviewing and providing feedback on our manuscript "Comparison of national and international sedentary behaviour and physical activity guidelines for older adults: A systematic review and quality appraisal with AGREE II". Revisions are noted as tracked changes within the manuscript and indicated below in italics under each reviewer's comments. The corresponding page and line numbers are associated with the track changed manuscript.

Reviewer 1:

1. Lines 71-71 - this sentence requires a quotation.

We have included quotes for the following: Sedentary behaviour is defined as "any waking behaviour characterized by an energy expenditure of 1.5 metabolic equivalents (METs) or lower while sitting, reclining, or lying", while sedentary time is measured by "the amount of time spent in these positions" (page 2, lines 49 to 51).

2. Lines 93-99 - Table S1 shows a fuzzy index database search! Why was the VoS database not used? A combination of keywords should be displayed for searching manuscripts

We consulted with two health science librarians from McMaster University and the University of British Columbia to review our search strategy; Web of Science does not have guidelines as a publication type but rather this database includes articles that discuss guidelines. Nevertheless, to address your point, we conducted another search in the Web of Science and CINAHL databases. We identified 231 studies in Web of Science, and 754 in CINAHL. We also conducted additional searches in Canada's Drug and health Technology Agency Grey Matters Database and the Scottish Intercollegiate Guideline Network. Through our new search, we identified six additional guidelines; we included two of the six guidelines and excluded the other four as they were not the latest version of the guideline.

We included the following statement in our manuscript: "*On August 22nd, 2023, we expanded our search to include Web of Science and CINAHL. We also searched for additional guidelines through Canada's Drug and health Technology Agency Grey Matters Database (https://www.cadth.ca/) and the Scottish Intercollegiate Guideline Network (https://www.sign.ac.uk/). A second librarian the University of British Columbia reviewed the search strategy*." (page 4, lines 105 to 109).

3. Lines 143-145 - These data do not agree with those in Figure 1

We updated Figure 1 and the manuscript, so it now reads: "*Our search strategy identified 44 clinical practice guidelines on sedentary behaviour and physical activity. We excluded*

26 guidelines as they were not the most recent version of the guideline, were not clinical practice guidelines, or were not available in English (Fig 1). We included 18 guidelines; ten of the 18 guidelines included sedentary behaviour recommendations (S2 Table) and all 18 guidelines include physical activity recommendations (S3 Table)." (page 6, lines 154 to 159).

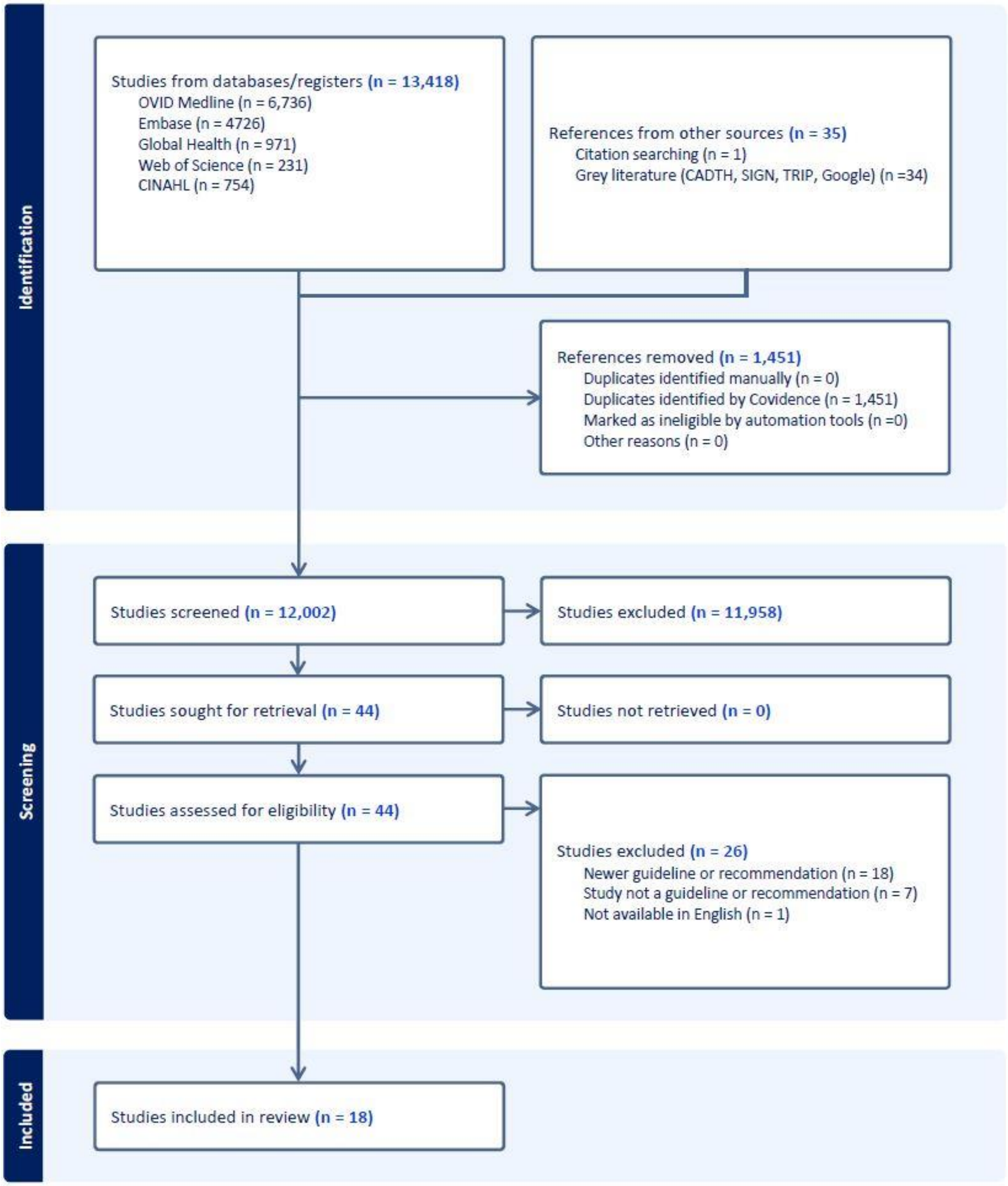

Fig 1. Identification, screening, eligibility, and included clinical practice guidelines.

4. Lines 257-262 - this text should be placed between Table 1 and Table 2

The following text was moved between Table 1 and Table 2: “*The guidelines published by the World Health Organization, USA, Germany, Canada, and Australia met the criteria for high quality, scoring at least 60% in at least 5 domains (Table 2). Guidelines published by Brazil, China, Netherlands, New Zealand, Norway, and United Kingdom met the criteria for average quality, scoring at least 60% in three to four domains. Guidelines published by Australia and New Zealand, Denmark, South India, Japan, Poland, and India had two or less domains that scored over 60% and were considered low quality*.” (page 12, lines 312 to 317).

5. Lines 381-383 - this has already been discussed, it should not be repeated. Here, conclusions should be given according to the author's opinion

We have removed the following text: “*Regarding the language limitation on the search strategy, we have exhausted all options in finding translated versions of guidelines. We were able to obtain a partial English copy of the Japanese guidelines.”*

Reviewer 2: I notice a lot of ambiguities:

1. Why does systematic review and quality assessment work at the same time? isn't that much on thay plate?

We agree that incorporating both a systematic review and quality assessment into a single manuscript is a significant undertaking; however, it is typical to conduct a systematic review and quality assessment at the same time. We followed the guidelines recommended by AGREE II, which was specifically designed to evaluate the methodological quality of practice guidelines during the review process (https://www.agreetrust.org/resource-centre/agree-ii/agree-ii-instructions/). Furthermore, previous PLOS One publication including "Methodological quality of clinical practice guidelines with physical activity recommendations for people diagnosed with cancer: A systematic critical appraisal using the AGREE II tool" (https://doi.org/10.1371/journal.pone.0214846) and “Assessing methodological quality of Russian clinical practice guidelines and introducing AGREE II instrument in Russia (https://doi.org/10.1371/journal.pone.0203328) have conducted systematic reviews and quality assessments at the same time.

2. I don't see a clear idea, but several ideas that bring confusion. It is difficult to follow the flow of research work. It is true that insight into best practices helps avoid duplication, and identify knowledge gaps. The authors mention it in Line 77, but later they have a completely different goal, and that goal is not very clear.

We have revised the wording to ensure clarity throughout the manuscript. Our updated statement is as follows: “*In the past decade, numerous countries have reviewed or updated their sedentary behaviour guidelines for older adults, with a trend toward more*

evidence-based guidelines [9–12]. A synthesis of international sedentary behaviour guidelines can help reveal more information about interventions that may be used to decrease sedentary behaviours, time spent engaging in these behaviours, and methods to decrease total sedentary time in older adults. In addition, most interventions have attempted to decrease sedentary time by increasing physical activity levels with the assumption that sedentary time would be reallocated to physical activity [13]. Therefore, reviewing physical activity guidelines can also provide insight into effective interventions that target sedentary behaviour since most sedentary behaviour guidelines are part of the physical activity guidelines [9–12]. Lastly, comparing sedentary behaviour and physical activity guidelines can provide insight into best practices, avoid duplication, and identify knowledge gaps. The purpose of this systematic review was to separately survey available sedentary behaviour and physical activity guidelines from around the world to compare recommendations and critically analyze the methodology through which the guidelines were developed." (page 3, line 71 to 91).

3. Why were Web of Science and Scopus not searched?

When we initially consulted with the librarian on our team, we chose not to use Web of Science or Scopus because these are not subject databases. Web of Science and Scopus do not have guidelines as a publication type, but rather, these database includes articles that discuss guidelines. We did not conduct a search in Scopus since the database publishes "primary document types from serial publications. Primary means that the author is identical to the research in charge of the presented findings"(https://www.elsevier.com/solutions/scopus/how-scopus- works/content). Guidelines are not a primary document. However, since Reviewer 1 also suggested we conduct our search in Web of Science we have updated our search strategy. This was our response to Reviewer 1:

"We consulted with two health science librarians from McMaster University and the University of British Columbia to review our search strategy; Web of Science does not have guidelines as a publication type but rather this database includes articles that discuss guidelines. Nevertheless, to address your point, we conducted another search in the Web of Science and CINAHL databases. We identified 231 studies in Web of Science, and 754 in CINAHL. We also conducted additional searches in Canada's Drug and health Technology Agency Grey Matters Database and the Scottish Intercollegiate Guideline Network. Through our new search, we identified six additional guidelines; we included two of the six guidelines and excluded the other four as they were not the latest version of the guideline.

We included the following statement in our manuscript: "*On August 22nd, 2023, we expanded our search to include Web of Science and CINAHL. We also searched for additional guidelines through Canada's Drug and health Technology Agency Grey Matters Database (https://www.cadth.ca/) and the Scottish Intercollegiate Guideline*

Network (https://www.sign.ac.uk/). A second librarian the University of British Columbia reviewed the search strategy." (page 4, lines 105 to 109)."

5. Why is a partial English copy of guidelines used?...

The full version of the Austrian guideline was not available in English. We were only able to locate a one-page document of the Austrian guideline that was written in English; the one page document was translated by the authors of the guideline. Thus, we were not able to critically appraise the quality of the guideline, but we were able to include the physical activity recommendations as they were translated to English. To address your concern, we have the following sentence: "*The full Austrian 2020 guideline was not available in English*" (page 12, line 309 to 310 and page 13, line 319 to 320).

The complete method is such that the research cannot be repeated. The author's obligation is to make it possible to repeat the same procedure. The structure is adequate. There are all the elements that the article should have. But the chapters themselves need to be improved. And the question is whether the idea is correct. But this manuscript does not have the strength required for a journal of Plos One quality.

We sincerely thank the reviewer for their insightful comments. We have carefully incorporated the reviewer's valuable feedback into our manuscript revisions and conducted a new search in Web of Science and CINHAL. Our librarian also helped search for additional guidelines in CDHT (Canada's Drug and Health Technology Agency Grey Matters Database) and SIGN (Scottish Intercollegiate Guideline Network). Through our search we identified two additional guidelines. We hope these changes align with Reviewer 2's expectations and kindly invite them to reconsider their assessment.

Reviewer 3: First of all, I can say that the authors have chosen a good topic, and the problem that was the subject of analysis in the following years will be more and more relevant, given that the average human life is longer and that more and more people are in the so-called third age. In that sense, the work has potential for readers. the work is methodologically well laid out, from the abstract to each individual part of the work. In the introduction. The work method is adequate, and as the authors themselves admit in the part of the limitations of the study, more relevant results would certainly have been obtained if the study sample had been larger and better, if recommendations from other speaking areas besides English had been included. Regardless, the results of the research, as well as the discussion that was done with quality, directed the authors to accurate and explicit conclusions. The references used are adequately listed with a fair number of younger ones. In any case, I recommend that the work be accepted.n, the key

terms related to the issue are well explained, and they are clearly supported by appropriate references.

Thank you for taking the time to read our manuscript and provide feedback. Your recognition of our chosen topic's relevance, methodological soundness, and quality of discussion is deeply appreciated. Your feedback encourages us to refine our work further. We are grateful for your recommendation and look forward to contributing meaningfully to the field.

Academic Editor: In addition to a point-by-point response to the comments from Reviewers #1, #2, and #3, I will highlight a few comments that are important to address. General Comments: When submitting your revision, we need you to address these additional requirements.

1. **Please ensure that your manuscript meets PLOS ONE's style requirements, including those for file naming. The PLOS ONE style templates can be found at**

https://journals.plos.org/plosone/s/file?id=wjVg/PLOSOne_formatting_sample_main_body.pdf and

https://journals.plos.org/plosone/s/file?id=ba62/PLOSOne_formatting_sample_title_authors_affiliations.pdf

We have changed the file name of our document to Main body as outlined in the https://journals.plos.org/plosone/s/file?id=wjVg/PLOSOne_formatting_sample_main_body.pdf example.

We have also changed the order of author affiliations to *Department, Institution, City, State, Country* as outlined in https://journals.plos.org/plosone/s/file?id=ba62/PLOSOne_formatting_sample_title_authors_affiliations.pdf.

2. **We note that the grant information you provided in the ‘Funding Information’ and ‘Financial Disclosure’ sections do not match. When you resubmit, please ensure that you provide the correct grant numbers for the awards you received for your study in the ‘Funding Information’ section.**

We have provided the correct information for the grants in the ‘Funding Information’ and ‘Financial Disclosure’ sections.

3. **Thank you for stating the following financial disclosure: "The funders had no role in study design, data collection and analysis, decision to publish, or**

preparation of the manuscript." At this time, please address the following queries:

a) Please clarify the sources of funding (financial or material support) for your study. List the grants or organizations that supported your study, including funding received from your institution.

We received financial support from the Hamilton Health Sciences New Investigators Fund, AGE-WELL-McMaster Institute for Research on Aging (MIRA) Award (page 22, lines 608 to 610).

b) State what role the funders took in the study. If the funders had no role in your study, please state: "The funders had no role in study design, data collection and analysis, decision to publish, or preparation of the manuscript."

We have included the following sentence in the manuscript: "*The funders had no role in study design, data collection and analysis, decision to publish, or preparation of the manuscript."* (page 22, lines 610 to 611).

c) If any authors received a salary from any of your funders, please state which authors and which funders.

We have included the following sentence in the manuscript: "IBR received a salary from the AGE-WELL-McMaster Institute for Research on Aging (MIRA) Award" (page 22, lines 611 to 613).

d) If you did not receive any funding for this study, please state: "The authors received no specific funding for this work."

N/A

Please include your amended statements within your cover letter; we will change the online submission form on your behalf.

4. We note that you have indicated that data from this study are available upon request. PLOS only allows data to be available upon request if there are legal or ethical restrictions on sharing data publicly. For more information on unacceptable data access restrictions, please see http://journals.plos.org/plosone/s/data-availability#loc-unacceptable-data-access-restrictions.

In your revised cover letter, please address the following prompts:

a) If there are ethical or legal restrictions on sharing a de-identified data set, please explain them in detail (e.g., data contain potentially sensitive information, data are owned by a third-party organization, etc.) and who has imposed them (e.g., an ethics committee). Please also provide contact information for a data access committee, ethics committee, or other institutional body to which data requests may be sent.

N/A

b) If there are no restrictions, please upload the minimal anonymized data set necessary to replicate your study findings as either Supporting Information files or to a stable, public repository and provide us with the relevant URLs, DOIs, or accession numbers. For a list of acceptable repositories, please see http://journals.plos.org/plosone/s/data-availability#loc-recommended-repositories.

We will update your Data Availability statement on your behalf to reflect the information you provide.

As this is a systematic review, there is no de-identified data set. All of the data that is necessary to replicate our study findings are available in the tables and figures provided. We are referring to:

S1 Table. Search strategy.

S2 Table. Summary of the sedentary behaviour guidelines.

S3 Table. Summary of the physical activity guidelines.

S4 Table. PRISMA 2020 Checklist.

Fig 1. Identification, screening, eligibility, and included clinical practice guidelines.

5. We note that this manuscript is a systematic review or meta-analysis; our author guidelines therefore require that you use PRISMA guidance to help improve reporting quality of this type of study. Please upload copies of the completed PRISMA checklist as Supporting Information with a file name "PRISMA checklist".

We have updated the document *S4 Table. PRISMA 2020 Checklist* to account for the changes in the clean version (i.e., Main body_clean).
